# Dual-Stream Fusion of Eye-Tracking and ECG Signals for Fatigue Detection in Remote Tower Air Traffic Controllers

**DOI:** 10.3390/bioengineering13070717

**Published:** 2026-06-23

**Authors:** Dajiang Song, Weijun Pan, Hugo Gamboa, Zirui Yin, Shengjie Wang

**Affiliations:** 1 Key Laboratory of Flight Techniques and Flight Safety, Civil Aviation Flight University of China, Guanghan 618307, China; 17726549663@163.com (D.S.); wsj@cafuc.edu.cn (S.W.); 2 LIBPhys (Laboratory for Instrumentation, Biomedical Engineering and Radiation Physics), Department of Physics, NOVA School of Science and Technology, NOVA University Lisbon, Largo da Torre, 2829-516 Caparica, Portugal; hgamboa@fct.unl.pt; 3 School of Transportation and Logistics, Southwest Jiaotong University, Chengdu 611756, China; yin@my.swjtu.edu.cn

**Keywords:** fatigue detection, physiological signal fusion, eye tracking, electrocardiogram, heart rate variability, deep learning, cross-subject calibration, air traffic controllers

## Abstract

Fatigue detection in remote tower air traffic controllers is important for maintaining operational safety under sustained visual monitoring and high cognitive workload. This study proposes MFD-Net, a dual-stream multimodal fusion framework using eye-tracking and electrocardiogram (ECG) signals. The model separately encodes eye-tracking and ECG-derived temporal inputs, incorporates an ECG-derived RMSSD expert feature, and performs lightweight late fusion for fatigue-state classification. Under the mixed-subject random-window protocol, MFD-Net achieved an Accuracy of 85.20%, a Recall of 83.33%, and an AUC of 0.9337. Because overlapping windows from the same participant and scenario could appear in both training and test sets, this result should be interpreted as a potentially optimistic within-distribution estimate. Under the stricter zero-shot leave-one-subject-out (LOSO) protocol, performance decreased substantially, with an Accuracy of 70.95±21.59%, a Recall of 22.98±36.30%, and an AUC of 0.6025±0.2984. This low zero-shot Recall indicates limited subject-independent fatigue-detection capability. Lightweight target-subject calibration and sequential probability aggregation improved adaptation and temporal stability, although the calibration results should be interpreted cautiously because random target-subject windows were used for fine-tuning. These findings suggest that eye-tracking and ECG fusion are promising under controlled conditions, while practical deployment requires deployment-oriented calibration protocols, recall-oriented optimization, and further real-world validation.

## 1. Introduction

Air traffic control (ATC) is a critical component of the modern aviation transportation system and plays an essential role in maintaining operational safety. Under complex operating conditions, including remote tower operations, high-density traffic management, and irregular shift schedules, controllers are often exposed to prolonged cognitive workload and continuous visual monitoring, which may contribute to fatigue accumulation [1]. Fatigue can slow decision-making, reduce attention resources, and impair situation awareness (SA), thereby posing potential risks to operational safety [2]. Therefore, developing a continuous and low-intrusion fatigue detection approach is important for improving the safety and reliability of ATC human–machine systems [3].

Remote tower operations further strengthen this need. Compared with conventional tower control, remote tower control relies more heavily on screen-based monitoring, multi-view display scanning, camera-based surveillance, and integrated digital interfaces, while direct out-of-window visual cues are reduced. During low-traffic or routine monitoring periods, prolonged screen observation may also become monotonous and increase the risk of vigilance decline. These characteristics make eye-tracking and ECG signals particularly relevant: eye-tracking can reflect visual scanning, fixation behavior, pupil response, and attention allocation across displays, whereas ECG can provide complementary information about autonomic regulation during sustained monitoring [4].

Current fatigue detection studies commonly use physiological and behavioral signals, such as electroencephalography (EEG), eye tracking, and electrocardiography (ECG). Although EEG is widely used for cognitive-state assessment, its relatively complex setup and limited convenience may restrict long-duration operational use [5]. Eye-tracking and ECG signals are comparatively low-intrusion and suitable for continuous monitoring, but they are heterogeneous in sampling rate, temporal structure, statistical distribution, and physiological meaning. In addition, substantial inter-subject differences in baseline physiological status, visual behavior, task strategy, and fatigue sensitivity make cross-subject generalization difficult. Class imbalance may further reduce model sensitivity to fatigue samples. Although multimodal fusion has been explored in previous studies, trade-offs remain among representation capability, model complexity, interpretability, and practical adaptability [6,7].

To address these issues, this study proposes a Multimodal Fusion Deep Network (MFD-Net) for fatigue detection in air traffic controllers using eye-tracking and ECG signals. The model extracts modality-specific temporal representations through a dual-stream encoding structure and performs lightweight late fusion with an ECG-derived window-level expert feature. Specifically, one-dimensional convolutional neural networks (1D-CNNs) are used to capture local temporal patterns, bidirectional long short-term memory networks (Bi-LSTMs) are used to model within-window temporal dependencies, and a Temporal Attention Module is used to emphasize informative signal segments. In addition, the root mean square of successive differences (RMSSD) is incorporated as an ECG-derived expert feature to supplement deep temporal representations with compact cardiac variability information. The novelty of MFD-Net should therefore be understood as a combined task-specific design rather than as a single isolated module. Its methodological contribution lies mainly in integrating modality-separated temporal encoding, ECG-derived RMSSD expert-feature injection, and lightweight late fusion for remote tower ATC fatigue detection. The LOSO calibration analysis and sequential probability aggregation are further used to evaluate cross-subject adaptability and temporal decision stability, rather than being presented as independent network-architecture innovations.

The main contributions of this study are summarized as follows:

1. A task-specific dual-stream multimodal framework is developed for fatigue detection in remote tower air traffic controllers. Instead of early concatenation of heterogeneous signals, eye-tracking and ECG data are encoded through separate temporal branches to preserve modality-specific visual-behavioral and cardiac physiological dynamics.
2. An ECG-derived RMSSD descriptor is incorporated as a window-level expert feature at the late-fusion stage. This design explicitly injects physiologically interpretable cardiac variability information into the deep multimodal representation, rather than treating all information as purely data-driven temporal features.
3. Beyond mixed-subject classification, the study systematically evaluates zero-shot LOSO generalization, lightweight target-subject calibration, sequential probability aggregation, RMSSD-window sensitivity, and weak-label sensitivity. These analyses clarify both the potential and the limitations of multimodal fatigue detection under cross-subject and low-latency monitoring conditions.

**Figure 1:**
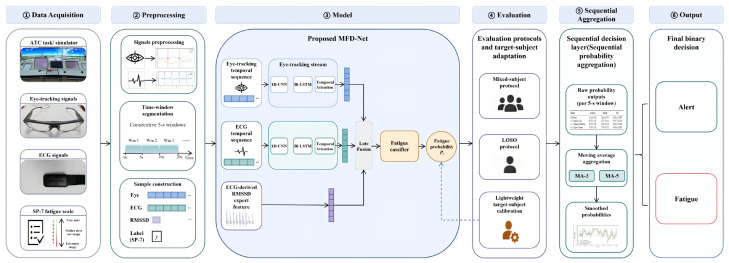
Overall technical workflow of the proposed MFD-Net framework for fatigue detection in air traffic controllers.

To provide an overview of the proposed framework, Figure 1 presents the main technical workflow of this study, from multimodal physiological data acquisition to fatigue-state classification.

The remainder of this paper is organized as follows. Section 2 reviews related studies on air traffic controller fatigue monitoring, physiological-signal-based fatigue detection, multimodal temporal modeling, and cross-subject adaptation. Section 3 presents the proposed MFD-Net method. Section 4 describes the experimental setup and data processing procedure. Section 5 reports the experimental results and analysis. Section 6 discusses the main findings, limitations, and future directions. Section 7 concludes the paper.

## 2. Related Work

### 2.1. Fatigue Monitoring and Cognitive-State Assessment in Air Traffic Control

In air traffic control (ATC), fatigue can be regarded as a multidimensional state of reduced cognitive–behavioral readiness caused by prolonged cognitive load, sustained visual monitoring, and continuous decision-making demands. It may manifest as reduced vigilance, slower information processing, impaired attention allocation, and decreased situation awareness [8,9]. This issue is particularly relevant to screen-based and remote tower operations, where controllers rely heavily on visual surveillance interfaces and multi-source information integration.

With the digital transformation of air traffic management systems, remote tower (rTWR) operations have progressed from early operational concepts to regulatory implementation [10]. Compared with conventional tower control based on direct out-of-window observation, remote tower control depends more strongly on screen-based surveillance, multi-view displays, and coordinated presentation of heterogeneous information sources. This operating mode places higher demands on sustained monitoring, visual search, target tracking, and information integration [11]. Therefore, fatigue in rTWR tasks is not only a subjective discomfort, but also a potential human-factor risk affecting situation awareness, response efficiency, and decision reliability.

Previous ATC-related physiological studies have mainly focused on workload, vigilance, and situation awareness rather than fatigue detection. For example, EEG and eye-tracking features have been used to identify changes in situation awareness under different workload levels in ATC tasks [12]. EEG-enabled Bayesian neural networks have also been applied to shared situation awareness assessment in adverse-weather aerodrome operations [13], and non-intrusive vigilance assessment methods have been proposed to reduce traffic-controller human-error risks [14]. These studies support the feasibility of physiological sensing in ATC scenarios, but fatigue detection and cross-subject adaptation remain less sufficiently investigated.

Recent studies have begun to examine fatigue in remote tower scenarios. Liang et al. modeled visual fatigue in remote tower air traffic controllers using multimodal physiological data and showed that visual behavior and physiological responses can jointly characterize fatigue-related changes [15]. Yin et al. proposed RTFnet, a multimodal physiological data fusion approach for fatigue detection in remote tower controllers [16]. These studies provide important evidence for remote-tower fatigue detection. However, existing methods have generally emphasized average classification performance, while the contribution of model components, cross-subject generalization, target-subject calibration, and temporal decision stability have not been sufficiently examined. This motivates further investigation of multimodal physiological fatigue detection in ATC tasks.

### 2.2. Physiological Signals for Fatigue Detection: Eye Tracking, ECG/HRV, and EEG

Subjective fatigue scales are simple and widely used, but they are intermittent and retrospective. In many fatigue-detection experiments, subjective ratings are collected before and after a task or scenario to avoid interrupting continuous performance [4,17]. As a result, labels are often available at the task or scenario level, whereas physiological signals are segmented into shorter windows for model training. Assigning the same subjective label to all short windows within a task segment is practical, but it creates coarse or weak labels because fatigue may fluctuate within the segment. This setting is conceptually related to weakly supervised learning or multiple-instance learning, where an episode-level label supervises multiple instance-level samples. Therefore, window-level fatigue classification based on scenario-level labels should be interpreted as detecting the dominant fatigue state of a task segment rather than precise moment-by-moment fatigue annotation.

Objective fatigue detection based on physiological and behavioral signals has become an important research direction in physiological computing and wearable human-state assessment [4,17]. Among low-intrusion modalities, eye tracking can characterize visual attention and cognitive-state changes through fixation, saccade, blink, pupil, and gaze-related measures [18,19]. Gaze entropy and eye-movement features can reflect changes in visual scanning under complex flight tasks [20], while blink frequency, blink duration, and PERCLOS are widely used for drowsiness and vigilance assessment [21,22].

ECG and heart rate variability (HRV) features provide fatigue-related information from the perspective of autonomic nervous system regulation. Forte et al. reported associations between HRV indices and executive control, attention, and cognitive flexibility [23]. Wearable fatigue-monitoring studies have also highlighted the value of ECG/HRV for long-term and low-intrusion fatigue assessment [17]. However, the interpretation of very short HRV windows should be cautious, as ultra-short-term HRV measures may not fully represent standard long-duration HRV characteristics [24].

EEG is sensitive to fatigue, vigilance, and workload changes. Borghini et al. reviewed neurophysiological measures for assessing mental workload, fatigue, and drowsiness in transportation tasks [3]. Dasari et al. found that ICA-derived EEG features were associated with mental fatigue, effort, and workload variations in a realistically simulated ATC task [25]. Ahn et al. further investigated fatigue-related neurophysiological changes using simultaneous EEG, ECG, and fNIRS data [26]. Despite its sensitivity, EEG usually requires more complex instrumentation and artifact control, which may limit its convenience for long-duration operational monitoring.

Overall, eye tracking and ECG/HRV provide a practical combination for ATC fatigue detection because they balance behavioral sensitivity, physiological interpretability, and low-intrusion acquisition. Eye tracking mainly reflects visual monitoring behavior and attention allocation, whereas ECG/HRV provides information related to autonomic regulation. Their complementarity suggests that joint eye-tracking and ECG modeling may provide a more balanced representation of fatigue-related changes than either modality alone.

### 2.3. Deep Temporal Modeling and Multimodal Fusion for Physiological Signals

Early fatigue detection studies often relied on handcrafted features and conventional classifiers, such as support vector machines, random forests, and shallow neural networks. These methods can be effective for structured small-sample problems, but they may be limited in modeling nonlinear, non-stationary, and temporally varying physiological signals. Deep learning provides a flexible route for physiological time-series classification. CNN-based models can extract local temporal or morphological patterns, whereas recurrent models can capture temporal dependencies in sequential signals [27].

Recent fatigue- and state-detection studies have increasingly used hybrid deep architectures. Barua et al. integrated EEG, EOG, and contextual information for driver sleepiness detection [28], while Mou et al. proposed an attention-based convolutional and recurrent multimodal fusion method for driver stress detection [29]. Kong et al. further developed a CNN–BiLSTM-based multimodal fatigue-detection framework using heart-rate-related signals and PERCLOS-based visual features [30]. These studies suggest that convolutional layers, recurrent structures, and attention mechanisms are useful for extracting discriminative representations from physiological and behavioral signals. Preserving temporal order is also important in time-series classification, especially under data scarcity and class imbalance [31].

Multimodal learning provides a general framework for integrating heterogeneous physiological signals. Baltrušaitis et al. summarized fusion strategies for heterogeneous data sources [6], and Ramachandram and Taylor reviewed deep multimodal learning for multisource information integration [7]. However, in physiological fatigue detection, more complex fusion mechanisms do not necessarily guarantee better generalization, particularly when the sample size is limited and inter-subject variability is substantial. Therefore, model design should balance representation capability, modality-specific temporal modeling, training stability, and interpretability.

Compared with RTFnet and related multimodal physiological models, MFD-Net does not aim to increase fusion complexity through a heavier cross-modal interaction structure. Instead, it adopts a lightweight task-specific design in which eye-tracking and ECG-derived temporal inputs are encoded through separate branches and then fused at a compact representation level. The ECG-derived RMSSD descriptor is incorporated as a window-level expert feature, providing physiologically interpretable cardiac variability information to supplement learned temporal representations. Thus, the contribution of MFD-Net lies in the combined design of modality-specific temporal modeling, expert-feature injection, and cross-subject evaluation for remote tower ATC fatigue detection.

### 2.4. Cross-Subject Generalization, Calibration, and Imbalanced Fatigue Recognition

Insufficient cross-subject generalization is one of the main obstacles to practical physiological fatigue detection. Physiological signals show substantial individual variability, including differences in baseline heart rate, HRV level, eye-movement patterns, fatigue expression, and responses to task demands. Therefore, a model may achieve high performance under mixed-subject random splitting but degrade when applied to unseen individuals. Chen et al. showed that inter-subject distribution differences can substantially affect driver-state detection [32], and Zeng et al. demonstrated the importance of transfer learning for cross-subject fatigue mental-state prediction [33]. These findings indicate that mixed-subject performance alone is insufficient to demonstrate true deployability in physiological fatigue detection.

Transfer learning, domain adaptation, and individual calibration are common strategies for reducing cross-subject distribution gaps. Ko et al. reviewed short- and zero-calibration approaches for EEG-based brain–computer interfaces [34], and Wu et al. summarized transfer learning methods for EEG-based brain–computer interfaces [35]. Although these studies mainly focused on EEG, the same principle is applicable to fatigue detection using eye-tracking and ECG signals. Accordingly, model evaluation should include strict subject-wise validation and lightweight target-subject calibration in addition to mixed-subject testing.

Cross-subject fatigue detection is also affected by class imbalance. Fatigue samples are often fewer than alert samples, while missed fatigue detection may be more critical than moderate false alarms in safety-related applications. Krawczyk pointed out that overall accuracy can mask poor minority-class recognition in imbalanced learning [36], and Iosifidis et al. showed that cost-sensitive boosting can improve imbalanced classification performance [37]. Therefore, fatigue detection models should be evaluated using Recall, F1-score, and AUC in addition to Accuracy.

Based on these observations, the present study focuses on three issues: improving fatigue representation through dual-stream eye-tracking and ECG temporal modeling, enhancing physiological information expression through an ECG-derived RMSSD expert feature, and examining adaptation to unseen subjects through LOSO evaluation and lightweight calibration. Sequential probability aggregation is further examined to assess whether window-level fatigue predictions can be made more temporally stable under continuous monitoring conditions.

## 3. Methodology

### 3.1. Overview of the MFD-Net Architecture

To address noise, nonlinear temporal variation, and inter-subject variability in multimodal physiological signals, this study proposes MFD-Net, a dual-stream multimodal fusion framework for fatigue detection in air traffic controllers. The framework uses eye-tracking and electrocardiogram (ECG) signals as the primary temporal inputs and further incorporates an ECG-derived RMSSD feature as a window-level expert descriptor. Through modality-separated temporal encoding and lightweight late fusion, MFD-Net jointly represents visual-behavioral and cardiac physiological information while preserving modality-specific characteristics.

**Figure 2:**
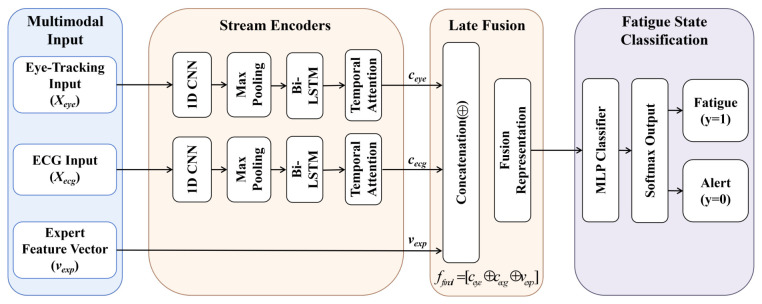
Overall architecture of MFD-Net for fatigue detection in air traffic controllers.

As shown in Figure 2, MFD-Net consists of four main components: multimodal inputs, dual-stream temporal encoders, a late-fusion module, and a fatigue-state classifier. The eye-tracking and ECG streams are processed by two independent temporal branches to account for their differences in sampling characteristics, temporal dynamics, and physiological meanings. The encoded representations are then concatenated with the RMSSD expert feature at the fusion stage and fed into the classifier to generate the final fatigue-state prediction.

It should be noted that the individual modules used in MFD-Net, such as 1D-CNN, Bi-LSTM, temporal attention, and RMSSD, are established techniques. The methodological focus of this study is therefore not the invention of a new basic neural-network module, but the task-specific integration of modality-separated temporal encoding, physiologically guided RMSSD expert-feature injection, and lightweight late fusion for low-latency fatigue detection in remote tower ATC tasks.

### 3.2. Problem Formulation and Input Definition

After preprocessing, including time-window segmentation, abnormal-segment removal, and normalization, fatigue detection was formulated as a multimodal binary classification problem. For each 5-s window, the model received three inputs: an eye-tracking temporal stream, an ECG-derived temporal stream, and an ECG-derived window-level expert feature. The output was the fatigue-state label y∈{0,1}, where y=1 denotes fatigue and y=0 denotes alert.

The eye-tracking input is denoted as $X_{eye}$

(1) $$X_{eye}=\left[x_{eye}^{\left(1\right)},x_{eye}^{\left(2\right)},\ldots ,x_{eye}^{\left(N_{eye}\right)}\right]\in R^{N_{eye}\times D_{eye}}$$

where $x_{eye}^{\left(t\right)}\in R^{D_{eye}}$ is the eye-tracking feature vector at time step *t*, $N_{eye}$ is the number of eye-tracking time steps within the window, and $D_{eye}$ is the feature dimension. This stream characterizes dynamic visual-monitoring behavior, including pupil-related and gaze-related temporal variations.

The ECG input is denoted as $X_{ecg}$

(2) $$X_{ecg}=\left[x_{ecg}^{\left(1\right)},x_{ecg}^{\left(2\right)},\ldots ,x_{ecg}^{\left(N_{ecg}\right)}\right]\in R^{N_{ecg}\times D_{ecg}}$$

where $x_{ecg}^{\left(t\right)}\in R^{D_{ecg}}$ is the ECG-derived cardiac temporal vector at time step *t*, $N_{ecg}$ is the number of ECG-derived time steps within the window, and $D_{ecg}$ is the feature dimension. It should be noted that $X_{ecg}$ does not represent the raw 512-Hz ECG waveform. The 512-Hz value refers to the acquisition rate of the ECG device. In the present implementation, the ECG branch used a one-channel ECG-derived cardiac temporal sequence constructed from the preprocessed ECG signal within each 5-s window. This stream was used to describe short-term cardiac temporal variations related to autonomic regulation.

In addition to the two temporal streams, an ECG-derived window-level expert feature was introduced. In the present implementation, this feature was RMSSD, which was computed from beat-to-beat interval differences within each 5-s ECG window. The expert feature vector is defined as

(3) $$v_{exp}\in R^{D_{exp}}$$

where $D_{exp}=1$. Unlike the temporal inputs, $v_{exp}$ is a scalar window-level descriptor and is injected at the late-fusion stage to supplement the ECG-derived temporal representation with compact cardiac variability information.

Accordingly, the multimodal input can be expressed as

(4) $$X=\left\{X_{eye},X_{ecg},v_{exp}\right\}$$

The specific variables included in the eye-tracking stream, the ECG-derived temporal stream, and the window-level expert feature are further described in the data preprocessing and sample construction section. Based on this input definition, the fatigue-state labels were used as supervision signals for model training, and the end-to-end mapping process is described in the following subsection.Based on this input definition, the fatigue-state labels were used as supervision signals for model training, and the end-to-end mapping process is described in the following subsection.

### 3.3. End-to-End Mapping

The overall architecture of MFD-Net follows a dual-stream parallel encoding and late-fusion design. Considering that eye-tracking and ECG signals differ in sampling characteristics, temporal patterns, and physiological response mechanisms, raw feature concatenation is not performed at the shallow stage. Instead, each modality is first processed by an independent temporal branch to extract high-level representations, which are then combined with the ECG-derived expert feature for final fusion and classification. Accordingly, the end-to-end mapping process of MFD-Net can be formulated as

(5) $$\hat{y}=F_{\mathrm{MFD}}\left(X_{eye},X_{ecg},v_{exp};\Theta \right)$$

where Θ denotes the set of learnable network parameters, and $\hat{y}$ denotes the predicted fatigue-state output.

Specifically, the mapping process first extracts high-level representations from the two modalities through dual-stream temporal encoders. The eye-tracking stream and the ECG stream are processed by their respective modality-specific encoders

(6) $$c_{eye}=\Phi_{eye}\left(X_{eye};\theta_{eye}\right)$$

(7) $$c_{ecg}=\Phi_{ecg}\left(X_{ecg};\theta_{ecg}\right)$$

where $\Phi_{eye}$ and $\Phi_{ecg}$ denote the eye-tracking and ECG encoding functions, respectively, and $\theta_{eye}$ and $\theta_{ecg}$ are the corresponding learnable parameters.

After obtaining the dual-stream high-level representations, the model combines the eye-tracking representation, the ECG representation, and the ECG-derived expert feature vector, and then outputs the fatigue-state prediction through the classifier. As shown in Equations (5)–(7), MFD-Net first uses parameter-independent dual-stream encoders to extract high-level representations from multimodal inputs and then completes the mapping from multimodal input to fatigue-state output through fusion and classification modules. This design preserves modality-specific characteristics while allowing complementary physiological information to be integrated within a unified framework.

### 3.4. Dual-Stream Temporal Encoding

To capture modality-specific temporal dynamics and physiological response mechanisms, a dual-stream temporal encoding structure is adopted for the eye-tracking and ECG inputs. Each stream consists of a one-dimensional convolutional neural network (1D-CNN), a max-pooling layer, and a bidirectional long short-term memory network (Bi-LSTM). The 1D-CNN layer is used to extract local sequential patterns within each 5-s window, such as short-term variations in gaze, pupil, or cardiac signals. The Bi-LSTM layer further models forward and backward temporal dependencies within the same window, allowing local physiological changes to be interpreted in their surrounding temporal context. In this way, local temporal variations and within-window sequential dependencies are jointly represented before temporal attention and multimodal fusion.

For an arbitrary modality input $X^{m}\in R^{N\times D_{m}}$, where m∈{eye,ecg}, local temporal pattern extraction is first performed by a one-dimensional convolutional layer

(8) $$H_{cnn}^{m}=\sigma \left(\mathrm{Conv}1D\left(X^{m};W_{cnn}^{m},b_{cnn}^{m}\right)\right)$$

where $W_{cnn}^{m}$ and $b_{cnn}^{m}$ denote the convolutional weights and bias, respectively, and σ(·) denotes the nonlinear activation function. This step is used to extract local variation patterns within each modality, such as short-term pupil and gaze changes in the eye-tracking stream and local rhythmic variations in the ECG stream.

Based on the convolutional features, a max-pooling layer is introduced to downsample the temporal representations, reduce redundant information, and improve feature compactness

(9) $$H_{pool}^{m}=\mathrm{MaxPool}\left(H_{cnn}^{m}\right)$$

After pooling, the feature sequence is fed into a Bi-LSTM to model temporal dependencies in both forward and backward directions. For the *t*-th time step, the bidirectional hidden state is expressed as

(10) $$h_{m}^{\left(t\right)}=\left[{\vec{h}}_{m}^{\left(t\right)}⊕{\overset{\leftarrow }{h}}_{m}^{\left(t\right)}\right]$$

where ${\vec{h}}_{m}^{\left(t\right)}$ and ${\overset{\leftarrow }{h}}_{m}^{\left(t\right)}$ denote the forward and backward hidden states of the LSTM, respectively, and ⊕ denotes feature concatenation. Through bidirectional modeling, the network can use contextual information from both directions within the input window.

After obtaining the hidden-state sequence, a Temporal Attention Module is introduced to assign adaptive weights to different time steps. For the *t*-th time step, the attention score is defined as

(11) $$e_{m}^{\left(t\right)}=v_{m}^{T}\tanh\left(W_{m}h_{m}^{\left(t\right)}+b_{m}\right)$$

where $W_{m}$, $b_{m}$, and $v_{m}$ are learnable parameters in the attention module. The attention scores are then normalized into attention weights

(12) $$\alpha_{m}^{\left(t\right)}=\frac{\exp\left(e_{m}^{\left(t\right)}\right)}{\sum_{j=1}^{T_{m}}\exp\left(e_{m}^{\left(j\right)}\right)}$$

where $T_{m}$ denotes the temporal length of modality *m* after convolution and pooling. Based on the attention weights, the high-level representation of modality *m* is defined as

(13) $$c_{m}=\sum_{t=1}^{T_{m}}\alpha_{m}^{\left(t\right)}h_{m}^{\left(t\right)}$$

For the eye-tracking stream and the ECG stream, the final modality-specific representations $c_{eye}$ and $c_{ecg}$ are obtained and subsequently fused with the ECG-derived expert feature vector $v_{exp}$.

The dual-stream temporal encoding structure serves two purposes. First, the 1D-CNN and pooling layers extract local temporal patterns and compress redundant information. Second, the Bi-LSTM and Temporal Attention Module model temporal dependencies and emphasize relatively informative time segments. Through parameter-independent branches, the model learns temporal representations of the eye-tracking and ECG modalities separately before multimodal fusion.

### 3.5. ECG-Derived Expert Feature Injection and Late Fusion

In this study, the window-level expert feature refers to the ECG-derived RMSSD extracted from each valid 5-s ECG window. RMSSD is not an independent modality and is not derived from eye-tracking data. Instead, it is an explicit and physiologically interpretable summary of beat-to-beat variability derived from the ECG signal. The eye-tracking information is represented separately by the eye-tracking temporal branch.

After dual-stream temporal encoding, the eye-tracking branch and ECG branch output the high-level representations $c_{eye}$ and $c_{ecg}$, respectively. The ECG temporal branch learns data-driven representations from the ECG-derived temporal sequence, whereas RMSSD directly summarizes successive beat-to-beat interval variation within the same window. Under short-window and limited-sample conditions, this variability information may not be fully or stably captured by the neural temporal encoder alone. Therefore, RMSSD is introduced as a compact window-level expert feature to provide a physiological inductive bias and to supplement, rather than replace, the learned ECG representation.

Instead of introducing an additional modality-gating mechanism, a direct late-fusion strategy is adopted. The two high-level temporal representations and the ECG-derived expert feature are concatenated to form a unified fused representation

(14) $$f_{final}=\left[c_{eye}⊕c_{ecg}⊕v_{exp}\right],$$

where ⊕ denotes feature-level concatenation, and $f_{final}$ denotes the final fused representation.

Direct late fusion was adopted, considering the heterogeneity of eye-tracking and ECG signals and the limited sample size of the present physiological dataset. The two modalities differ in sampling rate, temporal structure, statistical distribution, and physiological meaning; therefore, early fusion at the raw-signal or shallow-feature level may weaken modality-specific temporal patterns and increase sensitivity to alignment errors. Although intermediate fusion and cross-modal attention can model stronger inter-modal interactions, they usually introduce additional parameters and may increase the risk of overfitting under strong inter-subject variability. Therefore, the proposed model first encodes each modality independently and then performs lightweight high-level fusion, with RMSSD injected at the same fusion stage as a compact ECG-derived expert feature.

### 3.6. Classification Objective and Optimization

After obtaining the fused representation $f_{final}$ in Equation (14), the model maps it through a multilayer perceptron to generate the fatigue-state prediction

(15) $$z=W_{cls}f_{final}+b_{cls}$$

where $W_{cls}$ and $b_{cls}$ denote the weight matrix and bias term of the classification layer, respectively. The logits are transformed into a class-probability distribution through the Softmax function

(16) $$\hat{p}=\mathrm{Softmax}\left(z\right)$$

where $\hat{p}=\left[{\hat{p}}_{0},{\hat{p}}_{1}\right]$ denotes the predicted probabilities of the alert state and the fatigue state, respectively. Based on this probability distribution, the final predicted class label $\hat{y}$ is given by

(17) $$\hat{y}=\arg\max_{k\in \{0,1\}}{\hat{p}}_{k}$$

Considering that the constructed dataset exhibits class imbalance between alert and fatigue samples, directly using the standard cross-entropy loss may bias the model toward the majority class and reduce its ability to identify fatigue states. To address this issue, a weighted cross-entropy loss is adopted for training

(18) $$L=-\sum_{k\in \{0,1\}}w_{k}y_{k}\log\left({\hat{p}}_{k}\right)$$

where $y_{k}$ denotes the one-hot encoded ground-truth label, ${\hat{p}}_{k}$ denotes the predicted probability of class *k*, and $w_{k}$ denotes the loss weight assigned to class *k*. By assigning a larger weight to the fatigue class, the model places greater emphasis on fatigue-sample recognition during training, thereby alleviating the bias caused by class imbalance.

The model parameters are optimized by minimizing the loss function in Equation (18). Parameter updating is implemented through backpropagation and gradient-based optimization. Overall, MFD-Net achieves end-to-end fatigue-state modeling through dual-stream temporal encoding, ECG-derived expert feature injection, late fusion, and a weighted classification objective.

### 3.7. Sequential Decision Layer for Continuous Fatigue Assessment

To improve the temporal stability of continuous fatigue assessment, a lightweight sequential decision layer is introduced after the Softmax output of MFD-Net. The original model predicts the fatigue state independently for each 5-s window. However, fatigue-related states are unlikely to fluctuate abruptly between adjacent short windows. Therefore, aggregating consecutive window-level probabilities can provide a smoother decision trajectory while preserving the low-latency characteristic of the 5-s window design.

For the *i*-th 5-s window, the fatigue probability predicted by MFD-Net is denoted as

(19) $$p_{i}=P\left(y_{i}=1|X_{i}\right)$$

where $y_{i}=1$ indicates the fatigue state. Given a causal aggregation length *K*, the smoothed fatigue probability is calculated as

(20) $${\bar{p}}_{i}^{\left(K\right)}=\frac{1}{\left|S_{i}^{\left(K\right)}\right|}\sum_{j\in S_{i}^{\left(K\right)}}p_{j}$$

where

(21) $$S_{i}^{\left(K\right)}=\left\{\max(1,i-K+1),\ldots ,i\right\}$$

In this study, K = 3 and K = 5 are evaluated, corresponding to moving-average aggregation over three and five successive probability outputs, respectively. Under the 5-s window and 2-s stride setting, these aggregation lengths cover approximately 9 s and 13 s of signal duration, respectively.

(22) $${\hat{y}}_{i}^{\left(K\right)}=\left\{\begin{array}{cc} 1, & {\bar{p}}_{i}^{\left(K\right)}\geq \tau ,\\ 0, & {\bar{p}}_{i}^{\left(K\right)}<\tau \end{array}\right.$$

where τ denotes the decision threshold and is set to 0.5. To preserve causal applicability and avoid information leakage, only the current and previous windows within the same continuous record are used for aggregation. Predictions are not smoothed across different subjects or experimental scenarios.

The sequential decision layer is non-parametric and does not introduce additional trainable parameters. It is therefore used as a post-processing layer rather than a replacement for cross-subject adaptation. By progressively aggregating the initial 5-s predictions as additional windows become available, this layer reduces short-term decision fluctuations and improves the temporal stability of fatigue-probability trajectories.

## 4. Experimental Setup

### 4.1. Participants

Participants were recruited from the School of Air Traffic Management at the Civil Aviation Flight University of China (CAFUC). To ensure task relevance, all participants had received ATC-related training or had professional ATC instructional experience. All participants met the Class I medical certification requirements specified in the Civil Aviation Administration of China (CAAC) regulations for civil aviation personnel, had normal or corrected-to-normal vision, and had no known cardiovascular or central nervous system disorders.

A total of 34 trained participants were included in the final analyzable dataset, comprising 6 senior ATC instructors and 28 controller trainees. The senior instructors had approximately 9000±500 h of control experience. The trainees had completed systematic simulator-based training, passed the corresponding assessments, and accumulated approximately 1000 h of controller training experience. Detailed demographic and state-related variables, including age, sex/gender, sleep history, chronotype, and prior fatigue level, were not systematically recorded in the final analyzable dataset; therefore, these variables were not included as covariates in the present analysis. The participant characteristics and dataset composition are summarized in Table 1.

To reduce the influence of external factors on physiological signal acquisition, participants were instructed to avoid caffeine and alcohol for 24 h before the experiment. They were informed that they could withdraw voluntarily at any stage. All participants completed the experimental tasks in a high-fidelity remote tower simulation environment. All procedures were conducted in accordance with the Declaration of Helsinki and relevant data privacy requirements. Ethical approval was obtained from the Ethics Committee of the Flight Technology and Flight Safety Research Base, Civil Aviation Flight University of China, and written informed consent was obtained from all participants before the experiment.

### 4.2. Experimental Paradigm and Data Collection

**Figure 3:**
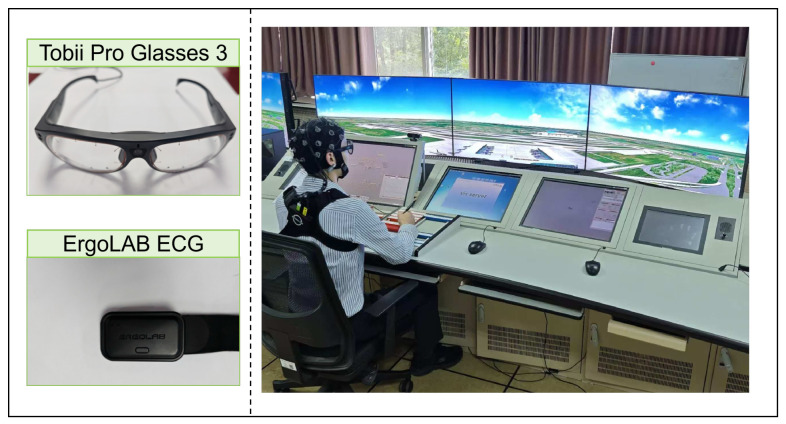
Experimental platform and physiological data acquisition devices. The right panel shows the high-fidelity Tower Client simulation platform used for the remote tower task, and the left panel shows the Tobii Pro Glasses 3 and the wearable ErgoLAB ECG device. The ECG device was worn on the chest. Other sensors visible in the setup were not included in the present analysis.

The experiment was conducted on a high-fidelity Tower Client simulation platform, as shown in Figure 3. The platform consisted mainly of a panoramic virtual tower visual display area, a controller operating terminal, and an instructor control terminal. The panoramic display presented external surveillance views of the runway and surface traffic, thereby simulating the screen-based external traffic perception mode in remote tower operations. The controller operating terminal was used by participants to perform the assigned control tasks, while the instructor control terminal was used for scenario configuration, procedure control, and experiment management.

During the simulated ATC tasks, eye-tracking and electrocardiogram (ECG) signals were recorded simultaneously. Eye-tracking data were collected using Tobii Pro Glasses 3 (Stockholm, Sweden) at a sampling rate of 100 Hz to capture fixation behavior, gaze shifts, and pupil variations during continuous visual monitoring. ECG data were collected using a wearable ErgoLAB ECG device at a sampling rate of 512 Hz for subsequent extraction of ECG-related and heart rate variability (HRV) features. The ECG device was worn on the chest. Although other sensors were present in the experimental setup, only eye-tracking and ECG data were analyzed in this study. The two signal streams were synchronously recorded during task execution and temporally aligned for subsequent analysis.

Subjective fatigue was assessed using the Samn–Perelli 7-point fatigue scale (SP-7) [38]. To avoid interrupting continuous task performance, SP-7 assessments were administered before and after each simulation scenario. In the subsequent analysis, the post-scenario SP-7 score was used as the scenario-level fatigue label for the physiological signal segments corresponding to that scenario. This label should be interpreted as a weak supervisory label representing the dominant fatigue state of the scenario, rather than as a precise moment-by-moment fatigue annotation for every 5-s window. This strategy was adopted because repeated subjective ratings during the control task would interrupt participants’ monitoring and decision-making processes and could alter the physiological responses being measured.

### 4.3. Data Preprocessing and Sample Construction

To construct the multimodal dataset, raw eye-tracking and ECG signals were processed using a unified window-based procedure. Continuous signals were segmented using a 5-s sliding window with a 2-s stride to support low-latency fatigue assessment while retaining short-term visual and cardiac response information. Because the two modalities were recorded at different sampling rates, synchronization was performed according to the unified task timestamp rather than at the raw-sample level. Within each 5-s window, modality-specific temporal inputs were constructed separately before fusion.

It should be clarified that the ECG temporal input used by MFD-Net was not the raw 512-Hz ECG waveform. The 512-Hz value refers to the acquisition rate of the ECG device. After preprocessing and artifact screening, cardiac temporal information was represented as a one-channel ECG-derived sequence within each 5-s window and used as $X_{ecg}$. RMSSD was computed separately from beat-to-beat interval differences and used only as an ultra-short-term ECG-derived cardiac variability descriptor and window-level expert feature, rather than as a standard long-duration HRV measure [24]. The use of 5-s RMSSD was motivated by the low-latency requirement of window-level fatigue detection. In this study, RMSSD was not interpreted as a conventional long-duration HRV measure, but was used as a compact ultra-short-term descriptor of beat-to-beat variability within the same prediction window. To reduce unreliable estimates, RMSSD was computed only for ECG windows with sufficient valid beat-to-beat interval information. Windows with missing R peaks, too few valid R–R intervals, or physiologically implausible beat-to-beat intervals were treated as unreliable for RMSSD estimation and excluded during ECG quality screening. A sensitivity analysis using longer causal RMSSD expert-feature windows was further conducted to examine the influence of this design choice.

To improve reproducibility, the input variables, sampling information, units, and preprocessing steps used for MFD-Net are summarized in Table 2.

During label mapping, the extreme-group approach was adopted to convert scenario-level SP-7 ratings into binary weak labels [39]. Samples with SP-7 scores of 1–3 were defined as the alert state (label 0), whereas samples with scores of 5–7 were defined as the fatigue state (label 1). Samples with a score of 4 were regarded as transitional states and excluded to reduce boundary ambiguity. Under this strategy, all valid 5-s windows within the same scenario shared the corresponding scenario-level label. Therefore, the labels reflect the dominant fatigue state of each scenario and may contain within-scenario label noise when short-term fluctuations occur.

After artifact removal, unreliable ECG-window exclusion, missing-signal screening, and label screening, 7766 valid 5-s multimodal samples were retained, including 5157 alert samples and 2609 fatigue samples. Because the fatigue class was relatively underrepresented, class imbalance was considered in subsequent model training.

### 4.4. Implementation Details

The proposed MFD-Net and all neural-network baseline models were implemented using the PyTorch deep learning framework (version 2.7.1+cpu; PyTorch Foundation, San Francisco, CA, USA). Model training and inference were performed on a local workstation.

During model training, the Adam optimizer was used to update the network parameters [40]. Based on preliminary experiments and initial hyperparameter search, the learning rate was set to $5\times 10^{-4}$, the batch size was set to 64, and the maximum number of training epochs was set to 40. To reduce the risk of overfitting under limited-sample conditions, dropout was introduced in the fully connected layers.

Considering the class imbalance of the constructed dataset, weighted cross-entropy loss was adopted for model optimization. The retained dataset contained 5157 alert samples and 2609 fatigue samples, corresponding to an alert-to-fatigue ratio of approximately 1.98:1. Therefore, the fatigue-class weight was initialized according to the inverse class-frequency tendency and then set slightly higher than the empirical imbalance ratio to reflect a safety-oriented preference for fatigue recall. Specifically, the alert and fatigue class weights were set to 1.0 and 2.5, respectively. This setting was used to penalize missed fatigue samples more strongly and to reduce the tendency of the model to favor the majority alert class. The same class-weight setting was kept fixed across the mixed-subject, LOSO, calibration, and ablation experiments, and was not tuned using the test data.

Unless otherwise specified, all models were trained and evaluated under the same experimental settings.

### 4.5. Evaluation Protocols, Metrics, and Baseline Models

To evaluate MFD-Net, both mixed-subject and subject-wise validation protocols were adopted. The mixed-subject random-window protocol divided the 7766 preprocessed samples into training and test sets at a ratio of 8:2, with stratified sampling used to maintain a similar class distribution. Because the samples were generated using overlapping 5-s windows with a 2-s stride, adjacent windows from the same participant and scenario could appear in both training and test sets. Therefore, this protocol was used only as a within-distribution reference and should be regarded as a potentially optimistic estimate rather than evidence of subject-independent or scenario-independent generalization.

To assess cross-subject generalization, a leave-one-subject-out (LOSO) protocol was employed. In each fold, one subject was held out, while the remaining subjects were used for training. In the lightweight calibration experiments, a small proportion of the held-out subject’s samples was additionally used for fine-tuning, and the remaining samples were used for testing. In the present implementation, target-subject calibration samples were selected randomly from the held-out subject while maintaining the class distribution where possible. This setting was used as an exploratory analysis of target-subject adaptability. However, because overlapping windows from the same subject and scenario could be split between calibration and testing, the calibrated LOSO results may also be optimistic and should not be interpreted as a fully deployment-realistic calibration protocol. A deployment-oriented design should use an earlier contiguous recording segment or a separate calibration scenario for subject-specific adaptation.

Model performance was evaluated using Accuracy, Precision, Recall, F1-score, and the area under the receiver operating characteristic curve (ROC-AUC). The classification metrics were calculated as follows:

(23) $$\mathrm{Accuracy}=\frac{TP+TN}{TP+TN+FP+FN}$$

(24) $$\mathrm{Precision}=\frac{TP}{TP+FP}$$

(25) $$\mathrm{Recall}=\frac{TP}{TP+FN}$$

(26) $$F1=2\times \frac{\mathrm{Precision}\times \mathrm{Recall}}{\mathrm{Precision}+\mathrm{Recall}}$$

where TP, TN, FP, and FN denote true positives, true negatives, false positives, and false negatives, respectively. ROC-AUC was used to assess model separability across decision thresholds. Considering class imbalance and the safety relevance of fatigue-sample recognition, Recall, F1-score, and ROC-AUC were emphasized in the result analysis.

For comparative evaluation, support vector machine (SVM) and random forest (RF) were selected as conventional machine-learning baselines. Two single-modality variants, namely Eye-only and ECG-only models, were also constructed to examine the contribution of each modality.

In addition to independent window-level evaluation, sequential decision aggregation was applied to the output probabilities of MFD-Net. Moving-average aggregation over 3 and 5 successive probability outputs was evaluated under the LOSO zero-shot and 30% fine-tuned settings. The aggregated outputs were assessed using the same classification metrics as the original 5-s predictions. Prediction switch rate and probability fluctuation were further calculated to quantify temporal stability during continuous fatigue assessment.

## 5. Results

### 5.1. Overall Performance Under the Mixed-Subject Random-Window Protocol

To provide a within-distribution reference, experiments were first conducted under the mixed-subject random-window protocol. After preprocessing, 7766 valid 5-s multimodal samples were used for training and testing. Because the samples were generated using a 5-s window with a 2-s stride, adjacent windows from the same participant and scenario could be randomly assigned to both the training and test sets. These overlapping windows may share signal segments and contextual information. Therefore, the results under this protocol should be interpreted as a potentially optimistic within-distribution estimate rather than evidence of subject-independent or scenario-independent generalization. Table 3 presents the comparison between MFD-Net and the baseline methods.

**Figure 4:**
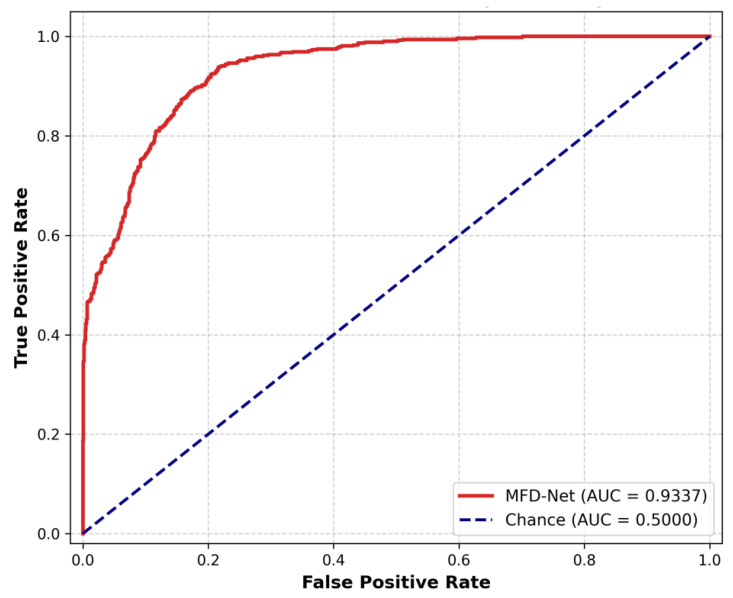
ROC curve of MFD-Net under the mixed-subject random-window protocol.

As shown in Table 3, MFD-Net achieved an Accuracy of 85.20%, a Recall of 83.33%, an F1-score of 79.09%, and an AUC of 0.9337. Compared with SVM and RF, MFD-Net showed a more balanced performance profile in this setting, especially in Recall and F1-score. However, this result should not be overemphasized as evidence of independent generalization. The stricter LOSO protocol and target-subject calibration analysis were therefore used as the main evidence for evaluating cross-subject generalization and adaptability. The ROC curve of MFD-Net under the mixed-subject random-window protocol is shown in Figure 4.

### 5.2. Single-Modality and Fusion Analysis

To examine the contribution of different input configurations, three model variants were compared: Eye-only, ECG-only, and All features (Fusion). The Eye-only model used only the eye-tracking temporal stream, the ECG-only model used only the ECG-derived temporal stream, and the All-features configuration corresponded to the complete MFD-Net input, including eye-tracking, ECG, and the ECG-derived RMSSD expert feature.

To further assess the stability of modality contributions, five-fold paired comparisons were conducted. Table 4 reports the mean performance and 95% confidence intervals of the three configurations.

**Figure 5:**
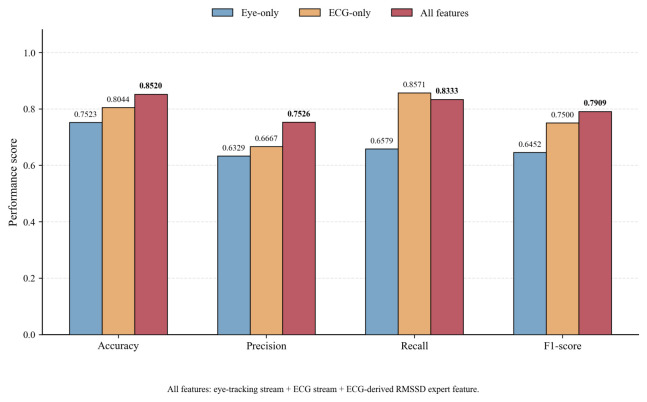
Performance comparison of different input configurations. All features denote the complete fusion configuration of MFD-Net, including eye-tracking, ECG, and the ECG-derived RMSSD expert feature.

As shown in Figure 5 and Table 4, the All-features configuration achieved higher Accuracy, Precision, F1-score, and AUC than both single-modality models. Compared with Eye-only, the mean improvements were 14.65 percentage points in Accuracy, 16.73 percentage points in F1-score, and 0.1325 in AUC. Compared with ECG-only, the corresponding improvements were 8.52 percentage points, 8.23 percentage points, and 0.0749, respectively. However, paired Wilcoxon signed-rank tests across the five folds did not reach the conventional significance threshold of 0.05 for these comparisons (p=0.0625). In terms of Recall, the All-features configuration was higher than Eye-only but lower than ECG-only. These results indicate that ECG contributed strongly to fatigue-sample recognition, whereas multimodal fusion mainly improved the overall balance among the evaluated metrics rather than achieving the highest value for every individual metric.

### 5.3. Sensitivity Analysis of RMSSD Expert-Feature Window Length

To examine the influence of RMSSD expert-feature window length, a sensitivity analysis was conducted while keeping the prediction window fixed at 5 s with a 2-s stride. The RMSSD expert feature was constructed using causal windows covering 5 s, approximately 11 s, and approximately 31 s, respectively.

As shown in Table 5, the longer causal RMSSD expert-feature windows did not produce consistent performance improvements. The 5-s setting achieved the highest Accuracy, F1-score, and AUC, while the approximately 11-s setting showed the highest Recall.

### 5.4. Sensitivity Analysis of Scenario-Level Weak Labels

Because fatigue labels were assigned at the scenario level, all valid 5-s windows within the same scenario inherited the corresponding post-scenario SP-7 label. This weak-label strategy may introduce label noise, particularly in fatigue-labeled scenarios where early windows may be assigned a fatigue label even though fatigue may have developed gradually later in the scenario. To examine the potential influence of this issue, a temporal-position sensitivity analysis was conducted by comparing three window-selection settings: all valid windows, the late 50% windows, and the late 33% windows within each scenario.

As shown in Table 6, using only the later portions of each scenario led to lower performance than using all valid windows, indicating that the scenario-level weak-label strategy may influence model training and evaluation. However, the late-window settings still maintained relatively high Recall and AUC values, suggesting that the main fatigue-related discrimination trend was not completely dependent on early-window samples. These results partly address the concern that early windows in fatigue-labeled scenarios may be mislabeled, but they do not eliminate the inherent limitation of scenario-level supervision. Therefore, the weak labels should be interpreted as practical scenario-level annotations of the dominant fatigue state rather than precise moment-level fatigue labels.

### 5.5. Cross-Subject Generalization and Target-Subject Calibration

To evaluate generalization to unseen individuals, a leave-one-subject-out (LOSO) protocol was adopted. In each fold, one subject was held out, while the remaining subjects were used for training. The fine-tuned setting denotes lightweight calibration using 30% of the held-out subject’s samples. In the present implementation, target-subject calibration samples were randomly selected while maintaining the class distribution where possible. Because overlapping windows from the same subject and scenario could be split between calibration and testing, the calibrated LOSO results should be interpreted as an exploratory and potentially optimistic estimate of target-subject adaptation rather than as a fully deployment-realistic calibration protocol.

As shown in Table 7, the zero-shot performance of MFD-Net decreased substantially compared with the mixed-subject setting. Recall decreased to 22.98±36.30%, indicating that the model missed many fatigue samples when directly applied to unseen subjects. Additional fold-level analysis also showed large variability in zero-shot LOSO performance, with Accuracy ranging from 0.00% to 100.00% and a mean of 75.24±29.05%. For held-out folds containing fatigue samples, Recall ranged from 0.00% to 99.51%, with a mean of 66.85±33.77%. These results indicate that zero-shot cross-subject fatigue recognition was highly unstable across held-out folds. Some folds contained only one class, which limited the interpretation of fold-level Recall, F1-score, and AUC. The large variability may be related to inter-subject physiological differences, class distribution differences, scenario characteristics, and scenario-level weak labels.

After lightweight calibration with 30% target-subject data, Recall increased from 22.98% to 40.16%, and Accuracy, F1-score, and AUC also improved. This suggests that target-subject information can help reduce the cross-subject distribution gap. However, because the calibration samples were randomly selected from overlapping target-subject windows, these results may still be optimistic. They should therefore be interpreted as evidence of potential target-subject adaptability rather than as a direct estimate of real deployment performance.

**Figure 6:**
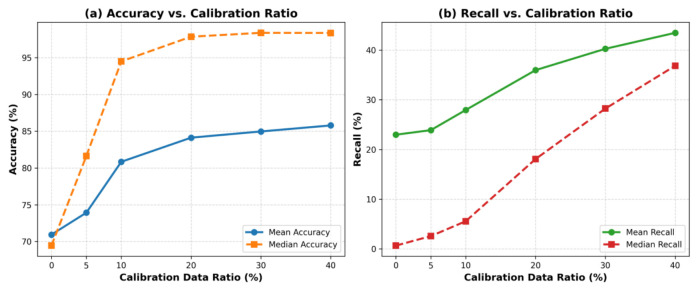
Sensitivity of MFD-Net to different target-data ratios under the LOSO protocol: (**a**) Accuracy versus calibration ratio; (**b**) Recall versus calibration ratio.

To further examine calibration sensitivity, MFD-Net was tested under calibration ratios of 0%, 5%, 10%, 20%, 30%, and 40%. The results are shown in Figure 6 and Table 8. Both mean and median values were reported because of the large inter-subject variability.

As shown in Figure 6 and Table 8, Accuracy and Recall generally increased as the calibration ratio increased. The 40% ratio yielded the highest mean Accuracy, Recall, and AUC, while the 30% ratio already provided a substantial improvement with less target-subject data. Therefore, the 30% ratio was used as the main lightweight calibration setting in subsequent analyses. The same random target-subject sampling strategy was used for all calibration ratios; thus, this analysis should also be regarded as an exploratory calibration-sensitivity estimate rather than a deployment-oriented chronological calibration evaluation.

### 5.6. Ablation and Visualization Analysis

A structural ablation study was conducted to examine the contribution of key components in MFD-Net, including the Temporal Attention Module, ECG-derived expert feature injection, and direct late fusion. Three variants were compared with the reference model: a modality-gating variant, a model without temporal attention, and a model without the ECG-derived expert feature. The experiments were performed under both mixed-subject 5-fold cross-validation and LOSO with 30% lightweight fine-tuning.

As shown in Table 9, the reference MFD-Net achieved the highest Accuracy, Recall, and AUC under the mixed-subject setting. Removing the ECG-derived expert feature caused the largest performance decrease, indicating that the RMSSD-based window-level feature provided useful cardiac variability information. This result should not be interpreted as evidence that RMSSD is independent of the ECG temporal stream. Rather, it suggests that explicitly providing beat-to-beat variability information helped the model, and that the ECG temporal branch alone may not have fully captured this information under short-window and limited-sample conditions. The modality-gating variant and the model without temporal attention also performed worse than the reference model, suggesting that the lightweight direct late-fusion design was sufficient for the present dataset.

Under the LOSO + 30% fine-tuning setting, Table 10 shows larger variability across folds. Removing the ECG-derived expert feature again led to a clear decrease in Accuracy and AUC, further supporting the usefulness of explicitly injected cardiac variability information. However, the large standard deviations indicate that the ablation results under cross-subject evaluation should be interpreted cautiously. The modality-gating variant did not outperform the reference MFD-Net, providing indirect support for the use of a lightweight direct late-fusion strategy. The variant without temporal attention achieved a higher AUC but slightly lower Accuracy and Recall, suggesting that the contribution of temporal attention was less stable under cross-subject evaluation.

**Figure 7:**
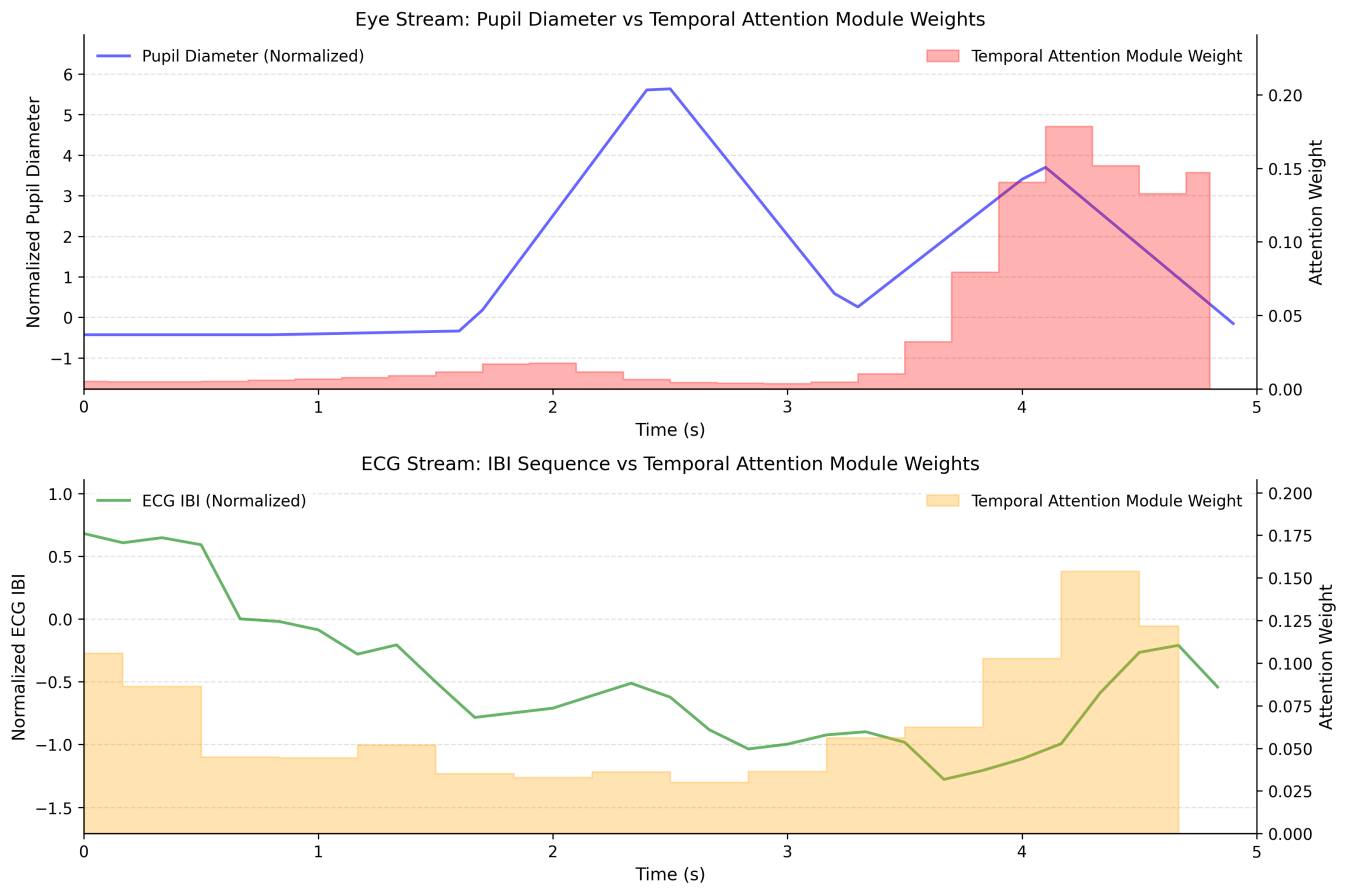
Visualization of dual-stream temporal responses of MFD-Net for a representative fatigue sample. The highlighted temporal responses indicate relative model emphasis within the sample and are intended only for qualitative interpretation; they should not be regarded as causal physiological evidence.

Two visualization analyses were further conducted as qualitative support. Figure 7 shows the dual-stream temporal responses of MFD-Net for a representative fatigue sample. The eye-tracking and ECG responses were not uniformly distributed across the 5-s window, but showed relatively higher weights in selected temporal segments. These weights only indicate relative model emphasis for this sample and should not be interpreted as causal physiological evidence.

**Figure 8:**
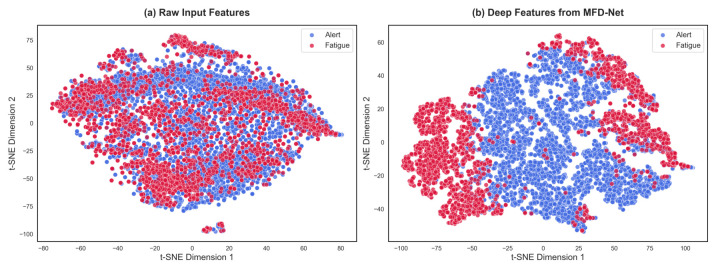
t-SNE visualization of raw input features and deep features extracted by MFD-Net. (**a**) Raw input features. (**b**) Deep features extracted by MFD-Net. Blue points indicate alert samples, and red points indicate fatigue samples.

Figure 8 presents the t-SNE visualization of raw input features and deep features extracted by MFD-Net. Compared with the raw feature space, the deep feature space showed a clearer separation tendency between alert and fatigue samples. Since t-SNE mainly reflects local neighborhood structure, this visualization is used only as supportive evidence and not as an independent measure of classification performance.

## 6. Discussion

### 6.1. Main Findings and Interpretation

This study investigated eye-tracking and ECG fusion for fatigue detection in air traffic controllers. Under the mixed-subject random-window protocol, MFD-Net achieved an Accuracy of 85.20%, a Recall of 83.33%, and an AUC of 0.9337. However, because overlapping 5-s windows from the same participant and scenario could appear in both the training and test sets, this result should be interpreted as a potentially optimistic within-distribution estimate rather than evidence of subject-independent or scenario-independent generalization. The single-modality analysis showed that the ECG-only model achieved the highest Recall, whereas the fusion model obtained better Accuracy, Precision, and F1-score. This suggests that ECG contains fatigue-sensitive information related to cardiac autonomic regulation, while eye tracking provides complementary behavioral information associated with visual monitoring and attention allocation.

The results provide cautious physiological insights into fatigue in remote tower ATC tasks. Fatigue-related changes appear to involve both visual-behavioral responses and cardiac autonomic regulation, rather than a single physiological source. The consistent contribution of the ECG-derived RMSSD expert feature suggests that beat-to-beat cardiac variability may provide useful fatigue-related cues during sustained monitoring. However, RMSSD should not be interpreted as information independent of the ECG temporal stream, because it is derived from the same ECG signal. Its contribution is better understood as the benefit of explicitly injecting a low-dimensional and physiologically meaningful cardiac variability descriptor. The strong degradation after removing RMSSD may indicate that the ECG temporal encoder alone did not fully or stably capture beat-to-beat variability information under short-window and limited-sample conditions.

The substantial performance degradation under zero-shot LOSO evaluation indicates that fatigue-related physiological manifestations are highly subject-dependent. Therefore, ATC fatigue detection should be regarded as a subject-sensitive physiological-state monitoring problem rather than a fixed universal pattern-recognition task. These interpretations remain classification-based and should not be treated as direct causal evidence of fatigue mechanisms.

The ablation results also clarify the methodological positioning of MFD-Net. The contribution of the proposed framework does not lie in the isolated use of eye tracking, ECG, temporal attention, or RMSSD. Instead, it lies in the task-specific integration of modality-separated temporal encoding, ECG-derived expert-feature injection, and lightweight late fusion for low-latency remote tower ATC fatigue monitoring. The modality-gating variant did not improve performance under the current data scale, suggesting that increasing fusion complexity does not necessarily lead to more stable performance in limited-sample physiological datasets.

Sequential probability aggregation improved the smoothness of window-level fatigue-probability outputs, but it could not overcome inter-subject variability under the zero-shot setting. Thus, sequential aggregation should be regarded as a post-processing strategy for improving temporal stability rather than a substitute for subject adaptation.

### 6.2. Comparison with Previous Studies

Compared with existing ATC-related studies, the present work focuses more directly on fatigue-state recognition. Previous studies mainly addressed situation awareness, workload, or collaborative monitoring in ATC and airport operational tasks [12,13]. Li et al. further proposed a non-intrusive vigilance assessment approach for reducing traffic-controller human-error risks [14]. These studies support the feasibility of physiological sensing for ATC cognitive-state assessment, whereas the present study further examines fatigue detection, modality contribution, and cross-subject adaptation.

Compared with remote tower fatigue studies, this work provides additional evaluation of model components and subject-wise performance. Liang et al. modeled visual fatigue in remote tower controllers using multimodal physiological data [15], and Yin et al. proposed a multimodal fusion method for fatigue detection in remote tower controllers [16]. Building on these studies, the present work compares single-modality and fusion configurations, conducts structural ablation, and evaluates the model under LOSO validation with lightweight calibration. These analyses help clarify not only the classification performance of the model, but also its sensitivity to unseen subjects.

From the perspective of modality selection, previous studies have shown that EEG is sensitive to fatigue, workload, and vigilance [3,25,26]. However, EEG acquisition usually requires more complex instrumentation and preparation, which may limit its convenience in long-duration operational monitoring. The present study instead uses eye-tracking and ECG signals, which are relatively low-intrusive and suitable for continuous task recording. This choice does not imply that eye-tracking and ECG are superior to EEG in all fatigue-detection tasks, but it provides a practical alternative when wearability and task compatibility are important considerations.

At the methodological level, this study is consistent with physiological computing research using CNN, recurrent networks, and attention mechanisms. Barua et al. and Mou et al. reported the usefulness of attention-based deep models in driver drowsiness and stress detection [28,29], and Rim et al. reviewed the broader application of deep learning in physiological signal analysis [27]. The present results support the relevance of temporal modeling for physiological fatigue detection, but also show that attention modules may not provide consistent gains across all validation protocols. This observation highlights the need to evaluate model components under both mixed-subject and subject-wise settings.

### 6.3. Cross-Subject Generalization, Calibration, and Deployment Feasibility

A key finding of this study is the clear gap between the mixed-subject random-window protocol and the stricter LOSO protocol. Under the mixed-subject protocol, MFD-Net showed relatively high performance; however, as noted earlier, this result may be optimistic because overlapping windows from the same participant and scenario could appear in both training and test sets. Under zero-shot LOSO evaluation, performance decreased to an Accuracy of 70.95±21.59%, a Recall of 22.98±36.30%, and an AUC of 0.6025±0.2984. This result is consistent with previous cross-subject studies showing that individual differences in physiological signals can weaken generalization to unseen subjects [32,33,34,35]. Therefore, mixed-subject performance alone is insufficient for evaluating fatigue-detection models intended for practical use.

The decrease in Recall is particularly important because missed fatigue cases are more critical than moderate false alarms in safety-related monitoring. The low zero-shot Recall suggests that fatigue-related patterns learned from the training subjects were not sufficiently stable across unseen individuals. This degradation may be related to inter-subject differences in baseline cardiac activity, gaze behavior, pupil response, fatigue sensitivity, class imbalance, and scenario-level weak labeling.

Lightweight target-subject calibration partially reduced the cross-subject gap. As the calibration ratio increased, Accuracy and Recall generally improved, and the 30% calibration setting provided a substantial proportion of the observed improvement. However, these calibrated results should be interpreted cautiously. In the present implementation, target-subject calibration samples were randomly selected from overlapping windows, so adjacent windows from the same subject and scenario could appear in both calibration and test subsets. Therefore, the calibrated LOSO results indicate the potential value of target-subject information, but they should not be regarded as a fully deployment-realistic calibration protocol. A more realistic calibration design should use an earlier contiguous recording segment or a separate calibration scenario before testing on subsequent data.

From a deployment perspective, the proposed system is not yet suitable for direct subject-independent use as a stand-alone fatigue alarm in real-world ATC environments. Instead, it should be regarded as a research prototype and a potential auxiliary monitoring tool. Practical use would require individual baseline collection, deployment-oriented calibration, recall-oriented threshold adjustment, periodic recalibration, and validation under real operational conditions. Any fatigue warning should also be interpreted within a human-in-the-loop safety management process rather than used as the sole basis for operational decisions.

### 6.4. Limitations and Future Directions

Several limitations should be noted. Although the experiment was conducted in a high-fidelity remote tower simulation environment, it could not fully reproduce the responsibility pressure, traffic uncertainty, long-duration workload accumulation, and organizational constraints of real ATC operations. Therefore, the present findings should be interpreted as evidence from a controlled simulation setting rather than direct proof of operational readiness.

The sample size was limited, and the distribution of professional experience was not fully balanced. The final dataset included 6 senior ATC instructors and 28 controller trainees, making separate instructor-versus-trainee modeling or statistical comparison underpowered and potentially unstable. In addition, detailed demographic and state-related variables, such as age, sex/gender, sleep history, chronotype, and prior fatigue level, were not systematically recorded in the final analyzable dataset. These factors may influence physiological fatigue responses and may partly contribute to the large inter-subject variability observed in the LOSO results.

Fatigue labels were derived from pre- and post-scenario SP-7 ratings; therefore, all valid 5-s windows within the same scenario shared the corresponding scenario-level label. This should not be interpreted as assuming that fatigue was strictly constant throughout the scenario. Instead, the label was used as a weak supervisory annotation representing the dominant fatigue state of the scenario. This strategy was practical for avoiding task interruption, but it may introduce label noise. In particular, early windows in a fatigue-labeled scenario may be assigned a fatigue label even if fatigue developed gradually later in the scenario. Although the late-window sensitivity analysis partly examined this issue, scenario-level labels still cannot fully capture short-term within-scenario fluctuations in fatigue state.

Although a sensitivity analysis using longer causal RMSSD expert-feature windows was added, RMSSD extracted from 5-s ECG windows should still be interpreted as an ultra-short-term ECG-derived cardiac variability descriptor rather than as a standard long-duration HRV measure [24]. The reliability of this descriptor depends on R-peak detection quality and the availability of sufficient valid beat-to-beat interval information within each window.

The lightweight target-subject calibration analysis also has limitations. In the present implementation, calibration samples were randomly selected from the held-out subject. Because the data were generated using overlapping 5-s windows with a 2-s stride, adjacent windows from the same subject and scenario may appear in both calibration and test subsets. Therefore, the calibrated LOSO results may be optimistic and should be interpreted as exploratory evidence of target-subject adaptability rather than as a fully deployment-realistic calibration protocol. Future deployment-oriented studies should use an earlier contiguous recording segment or a separate calibration scenario for subject-specific adaptation.

Future work should validate the proposed framework using larger and more diverse controller samples, longer-duration tasks, and more operationally realistic experimental settings. Future studies should also recruit a more balanced sample of instructors and trainees and record demographic, sleep-related, chronotype-related, and baseline fatigue information to examine how individual characteristics and experience level affect multimodal fatigue detection. More precise fatigue-labeling strategies, such as combining repeated subjective ratings, task-performance indicators, and continuous physiological trends, should be considered. In addition, more efficient cross-subject adaptation methods are needed to reduce the dependence on labeled target-subject data, and the sequential decision layer should be further evaluated in a real-time monitoring workflow, including decision thresholds, response delay, false-alarm management, human–machine interaction, and controller acceptance.

## 7. Conclusions

This study proposed MFD-Net, a dual-stream multimodal fusion framework for fatigue detection in air traffic controllers using eye-tracking and electrocardiogram (ECG) signals. The model integrates modality-specific temporal encoding, temporal attention, an ECG-derived RMSSD expert feature, and lightweight late fusion to jointly represent visual-behavioral and cardiac physiological information. Under the mixed-subject random-window protocol, MFD-Net achieved an Accuracy of 85.20%, a Recall of 83.33%, and an AUC of 0.9337. However, because overlapping windows from the same participant and scenario could appear in both training and test sets, this result should be interpreted as a potentially optimistic within-distribution estimate rather than evidence of independent generalization.

Under the stricter LOSO protocol, performance decreased substantially, especially in terms of Recall, indicating that direct subject-independent fatigue detection remains challenging. Lightweight target-subject calibration improved adaptation to unseen individuals, and moving-average aggregation helped smooth fatigue-probability outputs across consecutive windows. Nevertheless, the calibration results should also be interpreted cautiously because calibration samples were randomly selected from overlapping target-subject windows. Therefore, the proposed framework is more appropriately regarded as a calibrated auxiliary monitoring prototype rather than as a stand-alone fatigue alarm system for direct real-world deployment.

Future work should validate the proposed framework using larger and more balanced controller samples, longer-duration tasks, and more operationally realistic ATC settings. Demographic, sleep-related, chronotype-related, and baseline fatigue information should be recorded to examine individual differences in physiological fatigue responses. More precise fatigue-labeling strategies should also be considered by combining repeated subjective ratings, task-performance indicators, and continuous physiological trends. Methodologically, future studies should develop more robust cross-subject adaptation methods, use deployment-oriented calibration protocols based on earlier contiguous recordings or separate calibration scenarios, and further validate ultra-short-term ECG-derived features with stricter R-peak quality control. The sequential decision layer should also be tested in a real-time human-in-the-loop monitoring workflow, including decision thresholds, response delay, false-alarm management, and controller acceptance before practical deployment.

## Figures and Tables

**Table 1:** Summary of participant characteristics and dataset composition.

Group	Number	Experience or Training Background	Final Dataset Contribution
Senior ATC instructors	6	Approximately 9000±500 h of control experience.	Included in the final multimodal dataset after preprocessing and label screening.
Controller trainees	28	Completed systematic simulator-based training and corresponding assessments; approximately 1000 h of controller training experience.	Included in the final multimodal dataset after preprocessing and label screening.
Overall dataset	34	ATC-related trained participants recruited from CAFUC.	7766 valid samples in total, including 5157 alert samples (66.40%) and 2609 fatigue samples (33.60%).

**Table 2:** Summary of input variables, sampling information, units, and preprocessing steps used for MFD-Net.

Input Source	Sampling Information	Variables and Units Before Normalization	Preprocessing and Role in the Model
Eye-tracking temporal input	100 Hz acquisition rate	Seven-channel eye-tracking temporal input, including pupil-related and gaze-related variables. Original units included gaze coordinates and pupil diameter in mm.	Invalid or missing segments were removed. Signals were timestamp-aligned, segmented using a 5-s window with a 2-s stride, normalized using the training data, and used as $X_{eye}$.
ECG-derived temporal input	512 Hz acquisition rate; converted to a one-channel ECG-derived temporal sequence	One-channel cardiac temporal sequence derived from the preprocessed ECG signal within each 5-s window. This input was not the raw 512-Hz ECG waveform.	ECG segments with artifacts, missing values, or unreliable beat-to-beat information were removed. The ECG-derived sequence was timestamp-aligned with the corresponding eye-tracking window, normalized using the training data, and used as $X_{ecg}$.
ECG-derived expert feature	Derived from each valid 5-s ECG window	RMSSD extracted from valid beat-to-beat interval differences; the original unit was ms before normalization.	RMSSD was computed separately from the ECG-derived temporal stream, normalized using the training data, and injected at the late-fusion stage as the scalar expert feature $v_{exp}$.

**Table 3:** Performance comparison between MFD-Net and baseline algorithms under the mixed-subject random-window protocol.

Model	Accuracy	Precision	Recall	F1-Score	AUC
SVM	0.7246	0.5778	0.6272	0.6015	0.7646
RF	0.8230	0.8352	0.5806	0.6850	0.8894
MFD-Net	0.8520	0.7526	0.8333	0.7909	0.9337

**Table 4:** Five-fold performance comparison of single-modality and fusion configurations. Values are reported as mean ± standard deviation with 95% confidence intervals in brackets.

Model	Accuracy	Precision	Recall	F1-Score	AUC
Eye-only	73.45±2.01 [70.96, 75.94]	58.03±3.33 [53.90, 62.17]	78.27±4.79 [72.32, 84.22]	66.47±0.44 [65.92, 67.01]	0.8192±0.0055 [0.8124, 0.8260]
ECG-only	79.58±1.81 [77.33, 81.83]	63.85±2.57 [60.67, 67.04]	90.92±2.09 [88.32, 93.51]	74.97±1.54 [73.06, 76.89]	0.8769±0.0090 [0.8657, 0.8880]
All features (Fusion)	88.10±0.90 [86.98, 89.22]	79.41±3.82 [74.67, 84.16]	87.66±3.87 [82.86, 92.46]	83.20±0.91 [82.07, 84.33]	0.9518±0.0050 [0.9456, 0.9580]

**Table 5:** Sensitivity analysis of RMSSD expert-feature window length. The prediction window remained fixed at 5 s with a 2-s stride, and only the causal window used to construct the RMSSD expert feature was varied.

RMSSD Window	Approx. Span	Accuracy	Precision	Recall	F1-Score	AUC
5 s	5 s	0.8880	0.8187	0.8563	0.8371	0.9564
∼11 s	11 s	0.8629	0.7605	0.8640	0.8090	0.9304
∼31 s	31 s	0.8687	0.8354	0.7586	0.7952	0.9341

**Table 6:** Sensitivity analysis of scenario-level weak labels using different temporal portions of each scenario. Values are reported as mean ± standard deviation across repeated random splits.

Window Selection	Samples	Accuracy	Precision	Recall	F1-Score	AUC
All valid windows	7766	88.44±0.80	80.20±2.49	87.23±1.63	83.53±0.75	0.9506±0.0054
Late 50% windows	3885	82.71±0.39	72.25±2.63	79.18±4.36	75.45±0.61	0.8992±0.0108
Late 33% windows	2593	83.94±1.75	73.65±3.60	81.90±4.33	77.48±2.32	0.9066±0.0089

**Table 7:** Cross-subject performance comparison under the LOSO protocol.

Model	Setting	Accuracy	Precision	Recall	F1-Score	AUC
SVM	LOSO	62.49±16.01	28.37±27.96	19.65±27.76	19.03±24.35	0.5100±0.0800
RF	LOSO	69.46±24.50	25.70±29.70	12.47±26.97	11.39±23.46	0.4400±0.2000
MFD-Net	Zero-shot	70.95±21.59	41.08±42.56	22.98±36.30	24.25±35.59	0.6025±0.2984
MFD-Net	Fine-tuned	84.92±19.67	37.09±39.40	40.16±41.92	38.51±40.52	0.6497±0.2886

**Table 8:** Performance of MFD-Net under different target-data ratios in the LOSO protocol.

Ratio	Acc. Mean ± SD	Acc. Median (IQR)	Rec. Mean ± SD	Rec. Median (IQR)	AUC Mean ± SD
0%	70.95±21.59	69.45 (40.48)	22.98±36.30	0.66 (35.90)	0.6025±0.2984
5%	73.93±22.96	81.64 (47.93)	23.90±36.47	2.58 (30.96)	0.5509±0.3215
10%	80.84±21.67	94.49 (28.42)	27.95±37.59	5.57 (50.12)	0.6199±0.3040
20%	84.12±20.40	97.85 (25.45)	35.98±40.22	18.09 (68.24)	0.6329±0.3088
30%	84.97±20.00	98.37 (23.42)	40.27±41.83	28.24 (75.71)	0.6393±0.3017
40%	85.79±19.22	98.36 (22.77)	43.49±43.50	36.86 (82.85)	0.6506±0.2918

Note: Acc. = Accuracy; Rec. = Recall; SD = standard deviation; IQR = interquartile range.

**Table 9:** Structural ablation results under the mixed-subject 5-fold cross-validation setting.

Model	Accuracy	Recall	AUC
MFD-Net	83.44±1.69	89.38±3.00	0.9291±0.0087
w/ Modality Gate	80.71±0.87	87.77±1.10	0.9137±0.0115
w/o Temporal Attention	80.97±1.95	87.20±1.48	0.9111±0.0110
w/o Expert Feature	74.23±2.54	72.90±7.22	0.8156±0.0073

**Table 10:** Structural ablation results under the LOSO + 30% fine-tuning setting.

Model	Accuracy	Recall	AUC
MFD-Net	84.92±19.67	40.16±41.92	0.6497±0.2886
w/ Modality Gate	82.87±21.16	37.00±42.71	0.6460±0.2969
w/o Temporal Attention	84.71±20.10	39.85±42.46	0.7322±0.2388
w/o Expert Feature	67.38±22.85	37.92±36.75	0.5010±0.1319

## Data Availability

The data generated and analyzed in this study are not publicly available due to confidentiality agreements and privacy protection concerns for the participants.

## Abbreviations

The following abbreviations are used in this manuscript:

ATC	Air Traffic Control
rTWR	Remote Tower
ECG	Electrocardiogram
HRV	Heart Rate Variability
RMSSD	Root Mean Square of Successive Differences
EEG	Electroencephalography
SP-7	Samn–Perelli 7-point Fatigue Scale
MFD-Net	Multimodal Fusion Deep Network
CNN	Convolutional Neural Network
1D-CNN	One-Dimensional Convolutional Neural Network
Bi-LSTM	Bidirectional Long Short-Term Memory
LOSO	Leave-One-Subject-Out
SVM	Support Vector Machine
RF	Random Forest
AUC	Area Under the Receiver Operating Characteristic Curve
ROC	Receiver Operating Characteristic
MA	Moving Average
